# Comparative cranial biomechanics in two lizard species: impact of variation in cranial design

**DOI:** 10.1242/jeb.234831

**Published:** 2021-03-11

**Authors:** Hugo Dutel, Flora Gröning, Alana C. Sharp, Peter J. Watson, Anthony Herrel, Callum F. Ross, Marc E. H. Jones, Susan E. Evans, Michael J. Fagan

**Affiliations:** 1 School of Earth Sciences, University of Bristol, Bristol, BS8 1TQ, UK; 2 Department of Engineering, Medical and Biological Engineering Research Group, University of Hull, Hull, HU6 7RX, UK; 3 School of Medicine, Medical Sciences and Nutrition, University of Aberdeen, Aberdeen, AB25 2ZD, UK; 4 Institute of Life Course and Medical Sciences, University of Liverpool, Liverpool, L7 8TX, UK; 5 Centre for Integrative Anatomy, Research Department of Cell and Developmental Biology, University College London, Anatomy Building, Gower Street, London, WCIE 6BT, UK; 6 UMR 7179 MECADEV, MNHN – CNRS, Département Adaptations du Vivant, Muséum national d'Histoire naturelle, 75005 Paris, France; 7 Organismal Biology and Anatomy, University of Chicago, 1027 East 57th Street, Chicago, IL 60637, USA

**Keywords:** Lepidosauria, Squamata, Skull, Feeding, Finite element analysis, Multibody dynamic analysis

## Abstract

Cranial morphology in lepidosaurs is highly disparate and characterised by the frequent loss or reduction of bony elements. In varanids and geckos, the loss of the postorbital bar is associated with changes in skull shape, but the mechanical principles underlying this variation remain poorly understood. Here, we sought to determine how the overall cranial architecture and the presence of the postorbital bar relate to the loading and deformation of the cranial bones during biting in lepidosaurs. Using computer-based simulation techniques, we compared cranial biomechanics in the varanid *Varanus niloticus* and the teiid *Salvator merianae*, two large, active foragers. The overall strain magnitude and distribution across the cranium were similar in the two species, despite lower strain gradients in *V. niloticus*. In *S. merianae*, the postorbital bar is important for resistance of the cranium to feeding loads. The postorbital ligament, which in varanids partially replaces the postorbital bar, does not affect bone strain. Our results suggest that the reduction of the postorbital bar impaired neither biting performance nor the structural resistance of the cranium to feeding loads in *V. niloticus*. Differences in bone strain between the two species might reflect demands imposed by feeding and non-feeding functions on cranial shape. Beyond variation in cranial bone strain related to species-specific morphological differences, our results reveal that similar mechanical behaviour is shared by lizards with distinct cranial shapes. Contrary to the situation in mammals, the morphology of the circumorbital region, calvaria and palate appears to be important for withstanding high feeding loads in these lizards.

## Abstract (precis)

**Summary:** *In vivo* measurements and computer-based simulations of the cranial mechanics of two large lizards indicate that similar mechanical behaviour is shared by lizards with distinct cranial architecture, and show the importance of the postorbital bar in resisting the feeding loads.

## INTRODUCTION

Lepidosaurs, and particularly lizards (i.e. non-ophidian squamates), exhibit a remarkable anatomical and ecological diversity and have been used as a model to investigate the drivers of morphological and functional variation during evolution (Evans, 2008; Herrel et al., 2007; Stayton, 2006, 2008; Watanabe et al., 2019). The considerable diversity of skull forms in lizards has been well described (e.g. Evans, 2008; Watanabe et al., 2019), but comparative data on cranial biomechanics remains limited compared with that available in mammals. Unlike the mammalian skull, where the neurosensory organs are enclosed in a shell-like bony capsule, the skull of most lizards is an open framework of bars and struts. These architectural characteristics are likely to result in important differences in the mechanical behaviour of the cranium between mammals and lepidosaurs (Curtis et al., 2011a; Porro et al., 2014; Preuschoft and Witzel, 2002; Ross et al., 2018). Investigation of the biomechanics of the cranium in lepidosaurs thus provides an alternative perspective on skull function, which is important for formulating general principles regarding the factors driving skull shape diversity across tetrapods.

Previous studies have suggested that feeding behaviour and diet have a strong influence on the evolution of cranial shape in lizards (Herrel et al., 2007; McCurry et al., 2015; Metzger and Herrel, 2005; Stayton, 2011; Watanabe et al., 2019). This link probably reflects the response of the cranium to feeding loads in some ways, as the structural organisation and material properties of bones often vary to withstand muscle loads and external forces (Meakin et al., 2014). Yet, the skull performs many functions other than feeding, including housing and protecting the brain and sensory organs, supporting the respiratory tract, and providing ornaments for sexual display. Consequently, the evolution of a complex system such as the skull appears to be driven by diverse, and potentially conflicting, demands.

Data collected on other amniotes suggest that the overall shape of the skull is not optimally designed (i.e. maximum strength for minimum material) for resisting feeding loads (Hylander and Johnson, 1997; Hylander et al., 1991; Ross and Metzger, 2004). Therefore, bone shape and bone mass distribution in the cranium do not necessarily reflect an adaptation to feeding loads (Ross, 2001). In lepidosaurs, biomechanical simulations have demonstrated the importance of certain components of the skull, such as the lower temporal bar and the quadrate–pterygoid joint, in the structural resistance of the whole system (Moazen et al., 2009a,b; Wilken et al., 2019). By contrast, other features of the lepidosaur cranium appear to have no effect on its structural resistance to external loads. For instance, the chondrocranium has little influence on the strain regime of the surrounding cranial bones in the South American tegu (*Salvator merianae*) during simulated bites, suggesting that this structure serves to support the brain, eyes and olfactory organs rather than to absorb and redistribute feeding loads (Jones et al., 2017).

**Fig. 1.**
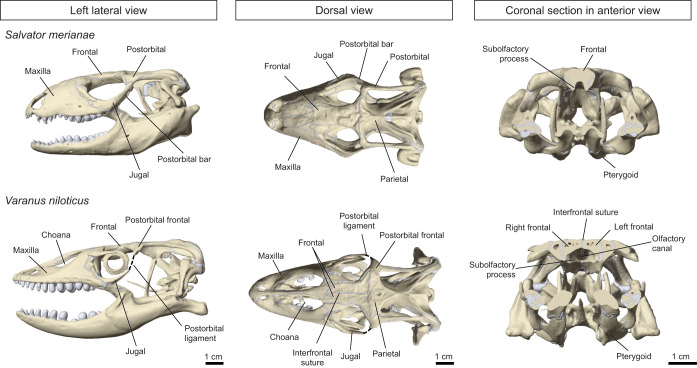
**Skull anatomy of *Salvatormerianae* and *Varanusniloticus*.**

The postorbital bar is formed by the dorsal extension of the jugal bone that connects to the postorbital or compound postorbitofrontal (Fig. 1) (Evans, 2008) and is present in most non-burrowing lizards, but has been reduced independently in two clades, Gekkota and Varanidae. In gekkotans, the postorbital bar and the supratemporal bar have been lost completely, with the jugal reduced to a small remnant in the ventral orbital margin. In varanids, the jugal is larger and extends roughly halfway up the postorbital margin, but it fails to meet the dorsal postorbitofrontal, leaving a gap of variable size. In gekkotans, loss of the bar has been linked primarily to constraints of space imposed by the increase in the size of the eye for nocturnal vision (Werner and Seifan, 2006), rather than a functional demand associated with feeding. Nonetheless, the loss of the postorbital bar has consequences for skull function during feeding by allowing a pronounced mesokinesis – movement of the snout relative to the postorbital region of the cranium – in different gecko species (*Gekko gecko*, *Phelsuma madagascariensis*, *Lialis burtoni*) (Herrel et al., 1999a, 2000, 2007; Montuelle and Williams, 2015; Patchell and Shine, 1986). The nature and amplitude of the intracranial movements are more contentious in varanids, and probably vary across species and during ontogeny (Metzger, 2002). Some varanid species (*Varanus bengalensis*, *Varanus exanthematicus*, *Varanus niloticus*) are reported to be mesokinetic (Frazzetta, 1962; Rieppel, 1978; Smith and Hylander, 1985), but others are probably not (Herrel et al., 2007; Metzger, 2002). Therefore, the presence or absence of a complete postorbital bar does not appear to be directly related to the pattern of intracranial kinesis in lizards.

How variation in cranial architecture and kinesis in lizards relates to the structural behaviour of the cranium in response to feeding loads remains unclear. It has been suggested that a complete postorbital bar increases the rigidity of the cranium by anchoring the sides of the snout to the back of the cranium (Evans, 2008; Jones et al., 2011; Porro et al., 2014; Ross et al., 2018). Therefore, the reduction of the postorbital bar might result in higher strain magnitudes in the cranial bones as a result of the bending of the snout during biting (Ross et al., 2018). Alternatively, taxa with intracranial kinesis might experience lower bone strain magnitudes, as feeding loads are dissipated by the more flexible components of the cranium (Ross et al., 2018). In this case, the structural integrity of the cranium would be preserved despite the reduction of the postorbital bar. In both varanids and gekkotans, the absence of a complete postorbital bar is also associated with an unusual frontal morphology in which the subolfactory laminae meet in the ventral midline (Fig. 1). The frontal plate forms a cylinder-like shape that might strengthen the skull while allowing the postorbital bar to be reduced (e.g. Evans, 2008). Comparison of *in vivo* strain gauge data obtained in different lizard species (Porro et al., 2014; Ross et al., 2018) suggests that variation in the distribution and magnitude of cranial bone strain is not obviously related to the degree of cranial kinesis, or the presence or absence of complete postorbital and supratemporal bars. As such, how the overall cranial shape and the postorbital bar, when present, affect strain magnitude and distribution in the cranial bones of lizards remains to be tested.

Computer-based biomechanical simulation techniques offer the opportunity to test *in silico* hypotheses of the function of biological structures. These approaches can further be used to create artificial morphologies (Gröning et al., 2013a; Lautenschlager et al., 2013; Moazen et al., 2009b; Nakashige et al., 2011; Sharp and Rich, 2016) and change the material properties of the tissues (Jones et al., 2017; Moazen et al., 2009a; Reed et al., 2011; Wilken et al., 2019) to assess the effect of a given structure in different scenarios. In the present study, we investigated cranial mechanics during feeding in two lizard species, the Argentine black and white tegu (*Salvator merianae* Duméril and Bibron 1839) and the African Nile monitor (*Varanus niloticus* Fitzinger 1826), by combining *in vivo* measurements with two *in silico* modelling techniques: multibody dynamic analysis (MDA) and finite element analysis (FEA). We used inverse dynamics in our MDA to calculate muscle activity, muscle forces, bite force and joint-reaction forces based on high-speed video recordings of the jaw movements during feeding. We then used the MDA results to define physiologically realistic boundary conditions for the FEA to calculate the strain pattern and magnitude generated by the feeding loads.

We chose the Argentine black and white tegu (*S. merianae*) and the Nile monitor (*V. niloticus*) as model organisms for the following reasons. First, both species are large (1–2 m in length) and, in the wild, are active, omnivorous hunters and scavengers. They both eat a wide variety of food materials including insects, eggs and small vertebrates, and both employ inertial feeding with larger prey items (Colli et al., 1998; Luiselli et al., 1999; Montuelle et al., 2009; Schaerlaeken et al., 2011). They thus occupy similar niches, albeit on different continents (South America, Africa). In fact, Daudin (1802) originally placed both species in the genus *Tupinambis*, although they are only distantly related (Tonini et al., 2016), and their respective lineages (Teiidae, Lacertoidea; Varanidae, Anguimorpha) diverged at least 150 million years ago (Burbrink et al., 2020; Pyron, 2017). *Salvator merianae* and *V. niloticus* display clear differences in their cranial shape and architecture, yet neither show any measurable mesokinesis (Herrel et al., 2007). The cranium of *S. merianae* is shallow and broad, with a short snout and a flat unpaired median frontal, whereas *V. niloticus* has a lighter, narrower cranium, an elongated snout, and a paired frontal with subolfactory laminae meeting in the ventral midline (Fig. 1). The frontals are separated along the midline by the interfrontal suture, which can fuse in old individuals. Importantly, a complete postorbital bar is present in *S. merianae*, but not in *V. niloticus*, where it is replaced dorsally by a short postorbital ligament (Fig. 1).

In this study, we used *S. merianae* and *V. niloticus* as model organisms to address the following questions. (1) Is the variation in cranial architecture observed between *S. merianae* and *V. niloticus* associated with differences in the loading regime (i.e. magnitude of bite force and muscle forces) of the cranium? (2) Are differences in the cranial architecture between *S. merianae* and *V. niloticus* related to differences in the deformation regimes of the cranial bones during biting? (3) More specifically, do the postorbital bar, present in *S. merianae*, and postorbital ligament, present in *V. niloticus*, have an impact on the pattern and magnitude of cranial bone strain? (4) Beyond obvious species-specific differences, are the overall deformation patterns of the cranial bones similar in these two species?

## MATERIALS AND METHODS

### *In vivo* bite force measurements and analyses

*In vivo* bite forces were measured on 63 wild and captive specimens of *S. merianae* and *V. niloticus* (Table S1). This sample includes the two specimens of *S. merianae* (ID: 000621516C) and *V. niloticus* (ID: 000617D5F1) used for biomechanical modelling (see below). The measurements were taken with a piezoelectric isometric Kistler force transducer (9311B; range ±5000 N) at the front of the jaw (Herrel et al., 1999b). The measurements at each bite position were repeated 5–10 times, and the highest measured force from those trials was retained as a measure for maximum bite performance.

We used multiple linear regression models to test the null hypothesis that mean bite force does not differ significantly between *S. merianae* and *V. niloticus*. We performed multiple linear regressions, each with one of the head dimensions (head width, head length and head depth) and the associated species as independent variables, and bite force as a dependent variable. All data were log_10_-transformed prior to statistical analyses, which were carried out in R (http://www.R-project.org/).

### Specimens used for biomechanical modelling

The specimen (ID: 000621516C) of the Argentinean black and white tegu *S. merianae* (formerly *Tupinambis merianae*) was an adult female with the following dimensions: snout–vent length (SVL) 360 mm, head length 80.05 mm, head width 56.73 mm, head depth 45.83 mm. The specimen (ID: 000617D5F1) of the Nile monitor *Varanus niloticus* (formerly *Varanus* *ornatus*; see Dowell et al., 2016) was an adult male with the following dimensions: SVL 435 mm, head length 81.07 mm, head width 44.34 mm, head depth 36.60 mm. Both animals were obtained through commercial dealers and housed in the Functional Morphology Laboratory, Department of Biology, University of Antwerp, Belgium, in conditions described by Ross et al. (2018). All experimental procedures were approved by the University of Antwerp Ethics Committee (reference 2006/18).

### High-speed video records and kinematic analysis

High-speed video records of the feeding events were made at the University of Antwerp, Belgium. The specimens of *S. merianae* and *V. niloticus* were filmed in lateral view while feeding. A Redlake Motion Pro 2000 digital high-speed camera (Integrated Design Tools, Inc., Tallahassee, FL, USA) attached to a Philips 14-inch image intensifier (Philips, Amsterdam, The Netherlands) was used to record the feeding events at 250 Hz. X-rays were generated using a Philips Optimus M200 X-ray generator (Montuelle et al., 2009; Schaerlaeken et al., 2011). The position of the tip of the upper and lower jaw was manually tracked in the software Tracker 5.1 (https://physlets.org/tracker/), and the gape (i.e. distance between the upper and lower jaw) calculated for each frame.

### Dissections

Animals were euthanised by an intramuscular injection of pentobarbital. The heads of *S. merianae* and *V. niloticus* specimens were dissected (from defrosted cadavers) and individual muscles separated. Muscles were immediately weighed after their dissection (wet mass), then placed into a 20% aqueous solution of nitric acid for 4–6 h to separate the individual muscle fibres. Nitric acid was replaced by a 50% aqueous solution of glycerol to stop the digestion, and 10–20 muscle fibres were randomly selected and photographed. The length of each fibre was then measured using the software Fiji (Schindelin et al., 2012) to calculate the average fibre length of each muscle (Table S2).

### Tomography, segmentation and mesh generation

Before dissection, the defrosted heads of the specimens were scanned at the University of Hull using X-Tek HMX 160 μCT system (Nikon, X-Tek Systems Ltd). The *S. merianae* head was scanned to obtain an isometric voxel size of 0.1112 mm with the following parameters: beryllium target, 113 kV, 25 μA, 1000 projections, 0.1 mm copper filter. The *V. niloticus* head was scanned to obtain an isometric voxel size of 0.1178 mm with the following parameters: 70 kV, 17 μA, 973 projections. After reconstruction, the image stacks were saved as .tiff files and imported in Avizo 9.2.0 (FEI Visualization Sciences Group, Hillsboro, OR, USA) for segmentation. For the finite element models, four materials were manually segmented based on their density: cortical bone, trabecular bone, sutures and teeth.

3D reconstructions of the skulls obtained from the segmentation were saved as .stl files and imported into Meshmixer (Autodesk, San Rafael, CA, USA) to be altered artificially. To test the role of the postorbital bar in cranial biomechanics, it was separated from the rest of the cranium in *S. merianae*, and digitally sculpted and inserted for *V. niloticus*. The artificial bar in *V. niloticus* had a surface area of 178 mm^2^ and a maximal cross-sectional area of 14.02 mm^2^. The dimensions of the artificial bar in *V. niloticus* were therefore similar to those of the *S. merianae* postorbital bar (surface area of 155 mm^2^, maximal cross-sectional area of 9.38 mm^2^). For both species, the postorbital bar was modelled as a separate segment from the rest of the cranium to test the effect of its presence and reduction on the cranial biomechanics by using the same mesh. For *V. niloticus*, the artificial postorbital bar was sculpted so that its extremities smoothly connected to the adjacent bones of the cranium. The surface of the postorbital bar was then imported into Avizo and converted into a 2D label that was added to the initial set of labels obtained from segmentation. This approach ensures that no artefacts are present at the boundaries between the artificial postorbital bar and the adjacent structures. The new set of labels was then used to generate a new 3D surface and then a finite element mesh of the cranium. In *V. niloticus*, the ventral lamina of the left and right frontal was separated from the rest of the bone and modelled as a separate material. Rendering of the surface models (Fig. 1) was performed in Blender 2.82 (https://www.blender.org/).

### Multibody dynamic analysis

MDA was performed in Adams 2015 (MSC Software, Newport Beach, CA, USA). The multibody dynamic models of *S. merianae* and *V. niloticus* comprised four and six moving parts, respectively. In both models, the cranium was fixed at the level of the foramen magnum, so that the other parts could move relative to it. In the *S. merianae* model, the two quadrates and the two hemi-mandibles moved independently and were connected to each other by different types of joints: the hemi-mandibles were connected at the mandibular symphysis by a spherical joint with 3 rotational degrees of freedom; the quadrate–mandibular joint was defined as a hinge joint with 1 rotational degree of freedom; the quadrato-squamosal joint was defined as a spherical joint with 3 rotational degrees of freedom.

In the *V. niloticus* model, the two quadrates, the two hemi-mandibles and the two pterygoids could move independently. The joints were modelled as follows: the hemi-mandibles were connected at the mandibular symphysis by a spherical joint with 3 rotational degrees of freedom; the quadrate–mandibular joint was defined as a hinge joint with 1 rotational degree of freedom; the quadrato-squamosal joint was defined as a spherical joint with 3 rotational degrees of freedom; the pterygoid-basipterygoid process joint was defined as a translational joint with 1 degree of freedom. For both models, the joint types and constraints were chosen based on the joint mobility assessed during the dissection of the modelled individual, and *in vivo* observations available for the same species. Moving parts were imported in Parasolid format to allow for the calculation of mass and inertial properties, the latter being calculated automatically in Adams 2015 using a bone density of 1.05 g cm^−3^ (Sellers and Crompton, 2004).

Muscles were discretised into a series of springs connecting their origin and insertion sites. When required, muscles were wrapped around the bone to represent the orientation of their line of action as accurately as possible. The physiological cross-section area (PCSA, in cm^2^) of each muscle was calculated using Eqn 1 (Sacks and Roy, 1982):(1)
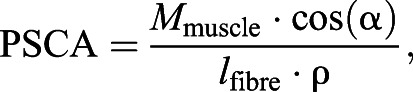
where *M*_muscle_ is the muscle mass (in g), α is the mean pennation angle of the muscle fibres (in deg), *l*_fibre_ is the mean fibre length (in cm) and ρ is the muscle fibre density (1.06 g cm^−3^) (Mendez and Keys, 1960). Maximum muscle force (*F*_max_, in N) was calculated based on the PCSA of the muscle, using Eqn 2 (Gans, 1982):(2)
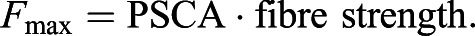


A maximum fibre strength of 40 N cm^−2^ was chosen for both species (Gröning et al., 2013a). The maximum muscle force was then divided by the number of strands representing the muscle in the multibody model and assigned to each strand of the muscle.

Inverse dynamic analysis was performed to calculate muscle force, the joint-reaction forces and the bite force based on kinematic data obtained from high-speed video records. The dynamic geometrical optimisation algorithm (Curtis et al., 2010) was used to simulate muscle activation dynamics during rigid-body motion. Muscle forces, joint-reaction forces and bite force corresponding to the maximum bite force for both anterior and posterior bite were exported in a format directly readable by the finite element software ANSYS v17 (ANSYS Inc., Canonsburg, PA, USA).

### Finite element analysis

The finite element meshes for *S. merianae* and *V. niloticus* consisted of 7,125,144 and 7,957,252 tetrahedral elements, respectively. Adaptive meshes were generated in Avizo to guarantee the modelling of small structures, such as the sutures, while limiting the number of elements and the size of the files. Meshes were then converted to .txt format using a custom-written R script (http://www.R-project.org/) and imported into ANSYS, where the linear 4-node tetrahedral elements were converted into higher-order 10-node tetrahedral elements (ANSYS SOLID 187). Bite force, muscle forces and joint-reaction forces calculated in the multibody model were then applied to the mesh. We chose to apply the joint-reaction forces calculated with the multibody model at the tip of the quadrate instead of constraining it to avoid erroneous contact stresses in this region. The resultant sum of all applied forces was close to zero (<0.1 N), confirming equilibrium of the applied loading, but to prevent any rigid body motion of the finite element model, the neurocranium was constrained at three nodes, in all degrees of freedom, around the foramen magnum. We chose to constrain the neurocranium as it is the fixed component of the cranium in the MDA model, with respect to which the other bones are moving. FEA was run for two loading cases: an anterior bilateral bite, and a posterior unilateral bite located at roughly 70% of the out-lever length (Lappin and Jones, 2014).

Both models consisted of four materials all modelled as homogeneous, isotropic and linear elastic. Materials were assigned a Poisson's ratio of 0.3 and the following elastic modulus values: cortical bone, 17,000 MPa; trabecular bone, 560 MPa; teeth (dentine), 5500 MPa; sutures, 20 MPa. Material properties were measured with a nanoindenter (CSM Instruments S.A., Peseux, Switzerland) on the defrosted *V. niloticus* specimen. Because of the limitation in the scan resolution, and the computational power needed to mesh the models, sutures were artificially enlarged but remained less than 0.4 mm thick. In varanids, the anterodorsal margin of the temporal fascia is thickened and forms the postorbital ligament spanning between the postorbitofrontal and the jugal (Fig. 1). We simplified this complex morphology and modelled the postorbital ligament with a 3D spring element (ANSYS LINK 180) spanning between the postorbitofrontal and the jugal. This spring element had uniaxial tension-only capability, and an assigned cross-sectional area of 2 mm^2^ based on measurements from the specimen used. Analyses were run for an elastic modulus of 50, 250 and 500 MPa and Poisson's ratio of 0.4. In the absence of data for lizards, we chose these values because they fall within the range of elastic modulus values reported for different ligaments in mammals (Munns et al., 1994; Nakagawa et al., 1996; Shetye et al., 2009; Stäubli et al., 1999; Vafek et al., 2018).

**Fig. 2.**
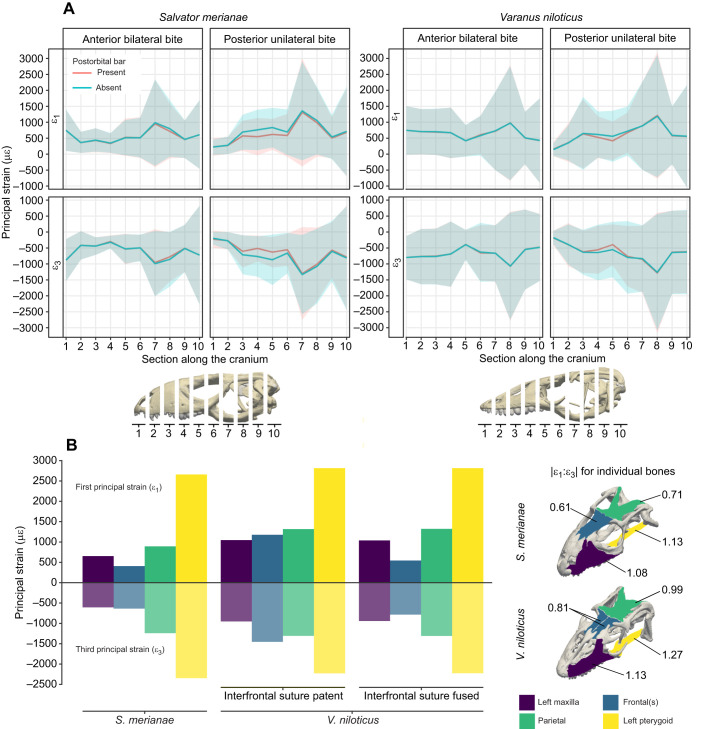
**Strain in the cranial bones of *S. merianae* and *V. niloticus*.** (A) Average first (ε_1_) and third (ε_3_) principal strain magnitude and standard deviation in 10 sections along the cranium. (B) Principal strain magnitude and corresponding |ε_1_:ε_3_| ratio averaged from the anterior bilateral and posterior unilateral maximal bite for selected bones. Strain magnitude is in microstrain (με).

When the FEA was completed, the first principal (ε_1_, most tensile) and third principal (ε_3_, most compressive) strains were exported from ANSYS. Quantitative analyses of the finite element results and post-processing to generate .vtk files were performed in R with custom-written scripts (http://www.R-project.org/). We used strain-based metrics because they have been demonstrated to better describe and predict the mechanical behaviour of bone than stress-based metrics (Fenech and Keaveny, 1999; Nalla et al., 2003; Schileo et al., 2008; Yosibash et al., 2010). Strain magnitude along the cranium was obtained (Fig. 2A; Table S3) by dividing the cranium into 10 sections of equal length and calculating the mean strain magnitude and standard deviation within each of those sections. Difference plots were used to visualise the effects of varying the model parameters on the magnitude and the distribution of the bone strain between the models. For each element, the relative strain difference (RSD) between the reference geometry (ε_ref_, i.e. models with the postorbital bar) and the alternative geometry (ε_alt_, i.e. models without the postorbital bar) was calculated for the first and third principal strains using Eqn 3:(3)
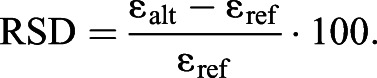

Rendering of the contour plots was performed in Paraview (Ahrens et al., 2005). Strain magnitude in individual bones was calculated by averaging the values collected from all the surface nodes forming the external surfaces of the bones (Tables S4 and S5). Comparisons were made between FEA results and *in vivo* strain gauge measurements published for *Anolis equestris*, *Iguana*, *Gekko gecko* and *S. merianae* (including the specimen here used for biomechanical modelling) (Ross et al., 2018). With respect to *S. merianae*, the average and maximal nodal strain recorded at each location of the strain gauge location were calculated from a series of analyses run with different loading conditions that replicated the *in vivo* transducer biting measurements published by Ross et al. (2018). The same was done with *V. niloticus* based on unpublished data that will be used for a future study. Maximal shear strain (γ_max_) at each gauge location was calculated by subtracting the first and third principal strain.

## RESULTS

### *In vivo* bite force, morphology and multibody dynamics results

Head dimensions were significant predictors of bite force (Table 1), and taxonomic group contributed significantly to the multiple linear regression model only when associated with head length. This probably reflects the difference in the relative length of the snout between the two species (Fig. 1). Our data thus support the null hypothesis that bite force does not differ between *V. niloticus* and *S. merianae*. The specimens selected for biomechanical modelling had similar maximal *in vivo* bite force magnitudes (Table 2), although *V. niloticus* showed a slightly higher bite force relative to skull width than *S. merianae*. The total adductor muscle mass (Table S2) was higher in *S. merianae* (24.90 g) than in *V. niloticus* (20.18 g), with the m. pterygoideus largely accounting for this difference, being about 1.6 times larger in *S. merianae*. When scaled to the same head width, the two species showed a similar total adductor muscle mass.

**Table 1.**
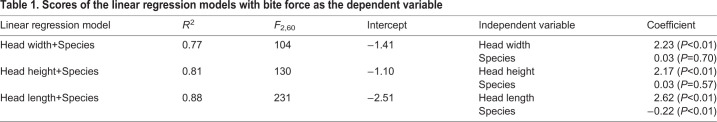
**Scores of the linear regression models with bite force as the dependent variable**

**Table 2.**
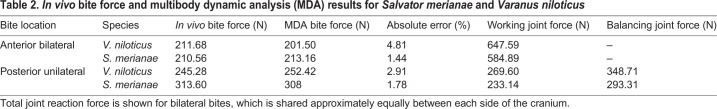
***In vivo* bite force and multibody dynamic analysis (MDA)** **results** **for *Salvator merianae* and *Varanus niloticus***

The MDA results are summarised in Table 2. The maximum bite force calculated with the MDA showed a good agreement with the *in vivo* bite force measurements collected for both species at the same location along the jaw, with less than 5% difference in each case. The geometry of the skull of *V. niloticus* appears to be better suited for transmitting muscle force to the bite point as its biting efficiency (i.e. bite force/total adductor muscle force) was 13% higher than in *S*. *merianae* during an anterior bite.

### Cranial biomechanics of *S*. *merianae* and *V. niloticus* under feeding loads

**Fig. 3.**
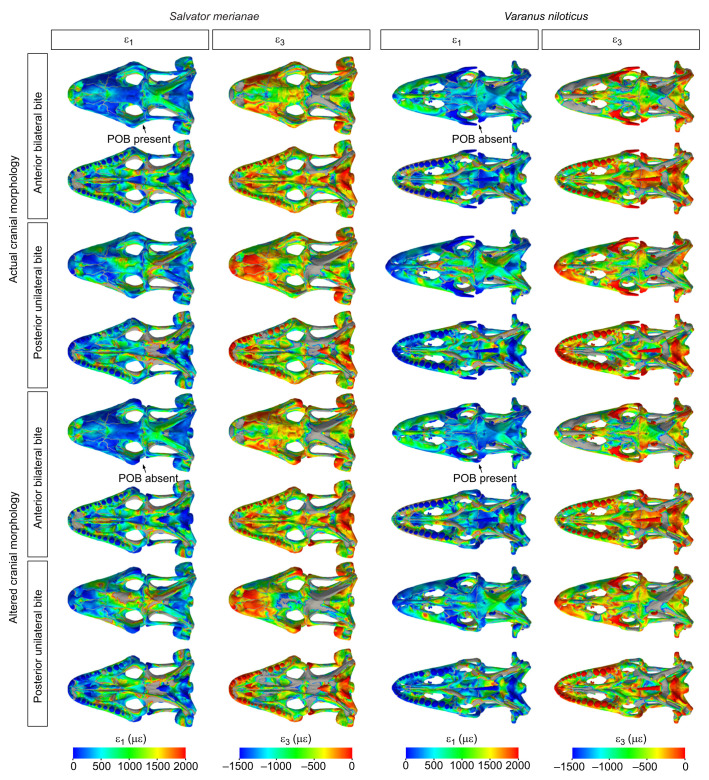
**Strain pattern in the cranium of *S. merianae* and *V. niloticus* and the impact of the postorbital bar (POB) on bone strain.** First (ε_1_) and third (ε_3_) principal strain calculated during an anterior bilateral and posterior unilateral bite. Results are presented for the actual and digitally altered cranial morphology (i.e. postorbital bar removed in *S. merianae* and added in *V. niloticus*). Strain magnitude is in microstrain (με); areas in grey correspond to out-of-range strain values.

The overall strain distribution and magnitude across the entire cranium was similar between the two species but notable differences were observed (Figs 2 and 3). Average bone strain magnitude across the cranium of *V. niloticus* was slightly greater during an anterior bite (Table S3). Differences in bone strain magnitude were, however, particularly marked in the snout (sections 1–4, Fig. 2A), where principal strain magnitude was 49% higher in *V. niloticus*, and between individual bones of the cranial roof (Fig. 2; Tables S3 and S4). The two species showed similar strain distribution across the cranium (Figs 2 and 3), with an anteroposterior gradient in tensile and compressive strain and greatest magnitudes in the posterior half of the cranium. Considering the individual bones, strain magnitude in the parietal was greater than in the maxilla, and the pterygoid experienced by far the greatest strain magnitude among the bones sampled in both species. The strain gradient was lower across the cranium and between the individual bones sampled in *V. niloticus* compared with *S. merianae* (Fig. 2). *Salvator merianae* experienced lower strain magnitude in its snout compared with *V. niloticus* and showed a sharper strain gradient between the antorbital–interorbital and postorbital regions of the cranium (sections 6–7, Fig. 2A). This was reflected in the strain magnitudes in the individual bones: strain in the frontal of *S. merianae* was about half that in the parietal, and tensile strain magnitude was 1.6 times lower in the frontal than in the maxilla, while compressive strain magnitudes were similar (Fig. 2B; Table S4). By contrast, in *V. niloticus*, strain magnitudes in the frontal were similar to those in the parietal and higher than in the maxilla. However, it is important to note that fusing the interfrontal suture in *V. niloticus* revealed a similar pattern between the two species: lower strain magnitudes in the frontal than in the parietal and the maxilla (Fig. 2B; Table S4).

The two species showed similar deformation regimes across the cranial bones sampled (Fig. 2B). Tensile strain, however, appeared more dominant (higher |ε_1_:ε_3_| ratios) in the cranial bones of *V. niloticus* (Fig. 2B). The maxilla and the pterygoid were predominantly under tensile strain (|ε_1_:ε_3_|>1; Fig. 2), whereas the frontal was mostly under compressive strain (|ε_1_:ε_3_|<1; Fig. 2). The parietal experienced predominantly compressive strain during an anterior bilateral bite in both species, and during a posterior unilateral bite in *S. merianae* only. The predominant tensile strain in the parietal of *V. niloticus* during a posterior bite resulted in a |ε_1_:ε_3_| ratio close to 1 when the two loading cases are averaged (Fig. 2B).

In both species, the greatest tensile strain (>2000 με) during an anterior bilateral bite was distributed in the bones forming the cranial floor (vomer, palatines, pterygoids) (Fig. 3). Peak compressive strain (<−1500 με) was in the premaxilla, prefrontal, parietal, the supratemporal bar, the pterygoids and palatines. *Varanus niloticus* differs from *S. merianae* in having large areas of compressive strain in the maxilla and on the lateral sides of the frontals, and greater strain magnitude in its elongated premaxilla and at the base of the parasphenoid. *Salvator* *merianae* displayed large peak compressive strain areas in the jugal and the postorbital bar.

During a posterior unilateral bite, the cranial floor of both species experienced greater tensile strain than the dorsal side of the cranium (Fig. 3). Dorsally, peak tensile strain in *S. merianae* was observed in the maxilla, in the frontal, the working-side supratemporal bar and in the contralateral side of the parietal, whereas in *V. niloticus*, peak tensile strain was solely located on the posterior half of the nasal and the left frontal. In both species, large compressive strain was found in the working-side antorbital arch, the balancing-side supratemporal bar, the contralateral side of the parietal and the working-side pterygoid. *Varanus* *niloticus*, however, displayed larger compressive strain areas in the prefrontal and in the working-side frontal. In *S. merianae*, the postorbital bar was predominantly under compression in all the loading cases simulated (Table 3). Compressive strain in the working-side postorbital bar was 2.5 times greater than on the balancing side during a posterior unilateral bite. Tensile strain in the left postorbital bar was, however, more dominant during an anterior bilateral bite than a posterior unilateral bite (Table 3).

**Table 3.**
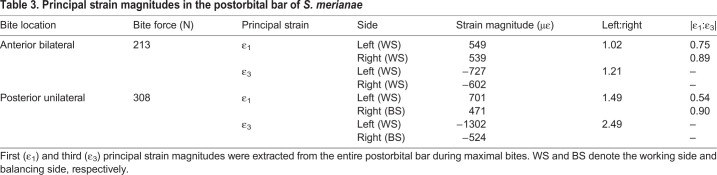
**Principal strain magnitudes in the postorbital bar of *S. merianae***

### Impact of the postorbital bar and ligament on bone strain

**Fig. 4.**
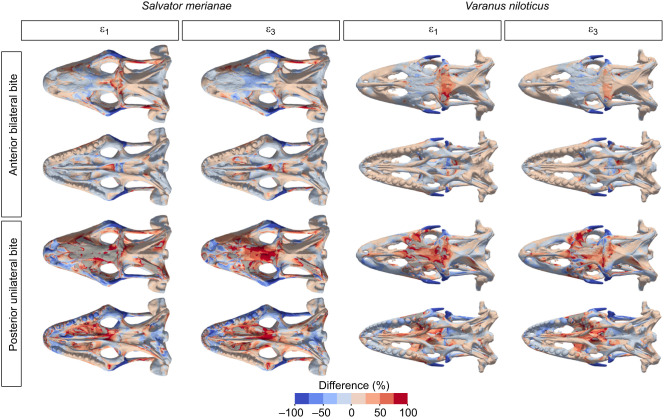
**Relative difference in principal strain between models with and without a postorbital bar.** Negative values (cold colours) correspond to higher strain when the postorbital bar is present, while positive values (warm colours) correspond to higher strain when the postorbital bar is absent. Areas in grey correspond to out-of-range strain values.

The effect of the postorbital bar on the magnitude and distribution of strain in the cranial bones was more marked in *S. merianae* than in *V. niloticus* (Figs 2–4). In both species, the impact of the postorbital bar on bone strain was more marked during a posterior loading case.

In *S. merianae* (Figs 2–4), removing the postorbital bar during an anterior bilateral bite increased absolute peak tensile and compressive strain in localised areas, such as the supratemporal bar (tensile strain) and the prefrontal (compressive strain). In the frontal, the absence of the complete postorbital bar resulted in an important increase (>50%) in tensile strain magnitude, and a decrease in compressive strain magnitude (Figs 3 and 4). This suggests that the postorbital bar is important for resisting bending during an anterior bite. Regions of the cranium (e.g. the snout) where low absolute strain values were recorded experienced a moderate increase in strain magnitude when the postorbital bar was added. Thus, the postorbital bar not only decreases peak strain but also redistributes strain over the whole cranium in *S. merianae*. The effect of the postorbital bar on cranial strain was clearer during the unilateral posterior biting case (Figs 2–4). Removing the postorbital bar increased the magnitude of tensile strain in the cranial roof bones by at least 75% as well as the size of high tensile strain regions in the left premaxilla and prefrontal, the frontal and the parietal (Figs 3 and 4). Areas of high compressive strain magnitude were larger in the prefrontal, frontal, parietal, postfrontal and palatine (Figs 3 and 4). On the balancing side, the premaxilla, prefrontal and palatine experienced much greater compressive strain when the postorbital bar was absent.

In *V. niloticus*, the impact of a complete postorbital bar on cranial bone strain was more limited than in *S. merianae* (Figs 2–4). During an anterior loading case, the addition of a complete postorbital bar reduced tensile strain magnitude in the anterior aspect of the parietal by more than 50%, and more moderately in the snout and the back of the skull (Figs 3 and 4). Compressive strain magnitude was also lower in the parietal, but slightly higher in the frontal when the postorbital bar was present (Fig. 4). During a posterior loading case, the inclusion of a complete postorbital bar clearly decreased tensile strain magnitude in the nasal, prefrontal, anterior aspect of the parietal in the dorsal skull, and left vomer, palatine and pterygoid ventrally (Figs 3 and 4). The inclusion of the postorbital bar also decreased compressive strain magnitude in the dorsal surface of the frontal and in its subolfactory process (Figs 3 and 4). This suggests that the frontal morphology in *V. niloticus* may increase the structural resistance of the cranial roof in the absence of a complete postorbital bar.

**Fig. 5.**
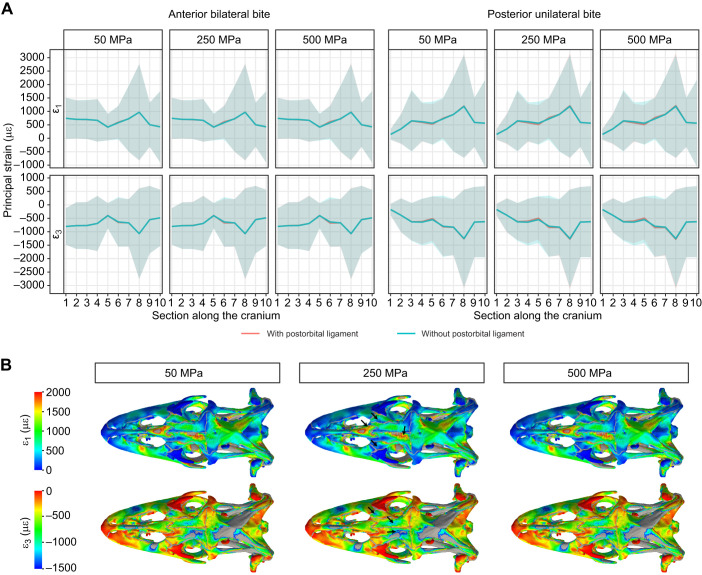
**Effect of the postorbital ligament on bone strain in *V. niloticus*.** (A) Average first (ε_1_) and third (ε_3_) principal strain value and standard deviation in 10 sections along the cranium calculated for different values of the Young's modulus of the postorbital ligament. (B) Contour plots in dorsal view showing the effect of varying the Young's modulus of the postorbital ligament on bone strain during a posterior unilateral bite. Areas in grey correspond to out-of-range strain values.

*Varanus niloticus* lacks a complete postorbital bar but a postorbital ligament spans the dorsolateral gap between the jugal and the postorbitofrontal (Fig. 1). The effect of the postorbital ligament on the overall strain in the skull is minor compared with that of the postorbital bar (Fig. 5A). Increasing the stiffness of the postorbital ligament slightly decreased peak tensile and compressive strain magnitude in the frontal, nasal and anterior parietal during a posterior bite (Fig. 5B). Therefore, it is unlikely that the postorbital ligament alone fulfils the mechanical role of a postorbital bar in *V. niloticus*.

### Impact of frontal shape on the cranial biomechanics of *V. niloticus*

**Fig. 6.**
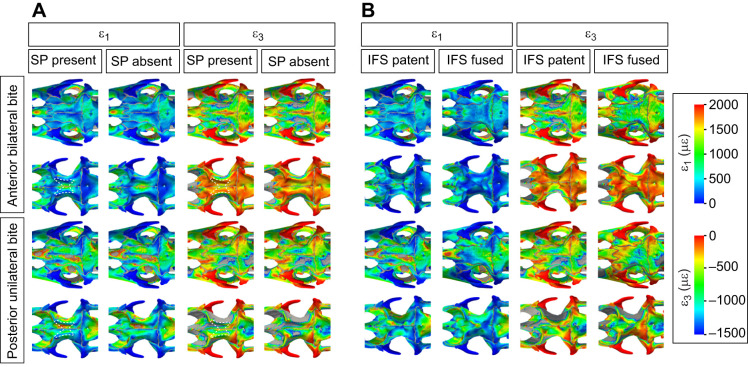
**Strain in the frontals of *V. niloticus*.** A dorsal view of the cranium and a ventral view of a transverse section of the cranial roof are shown. (A) Effect of the subolfactory process (SP) on strain in the frontal. The subolfactory processes are outlined when included in the analyses but hidden in the rendering to observe strain on the ventral side of the frontals. (B) Effect of the interfrontal suture (IFS) on bone strain. Areas in grey correspond to out-of-range strain values.

Altering the morphology of the frontal affected the strain magnitude in this bone but had a limited impact on the strain regime in the rest of the cranium (Fig. 6). Removing the subolfactory processes of the frontals markedly increased strain in the ventral surface of the frontal during a posterior bite (Fig. 6A), with larger peak tensile strain (>2000 με) areas on the working side frontal and larger peak compressive strain (<−1500 με) areas on its counterpart. The subolfactory processes of the frontals therefore appear to increase the structural resistance of the interorbital region of the cranial roof.

Fusing the interfrontal suture decreased tensile and compressive strain magnitude in the frontal of *V. niloticus* by about a half without really affecting the adjacent parietal (Figs 2 and 6B; Table S4), but strain magnitude remained higher than in the frontal of *S. merianae*. Strain was more evenly distributed in the frontals (Fig. 6B), and compressive strain became more dominant (|ε_1_:ε_3_|=0.69; Table S4). Notably, this is reflected during a posterior unilateral bite by large peak compressive strain in the balancing side subolfactory processes (Fig. 6B), which further highlights the importance of these structures for resisting feeding loads as the interfrontal suture can fuse in large adults.

## DISCUSSION

### Comparison and determinants of cranial bone strain in *V. niloticus* and *S. merianae*

We observed that bone strain magnitude was more uniform along the cranium in *V. niloticus* than in *S. merianae*. Strain magnitude was higher in the maxilla and other cranial bones of *V. niloticus* and in the entire anterior portion of the cranium (Fig. 2; Table S3). These differences were irrespective of the presence of a complete postorbital bar or postorbital ligament (Figs 2–5), but were most probably linked to the distinct snout form observed in these two species. Previous FEA predicted that archosaurs with fenestrated and flattened snouts (i.e. platyrostral cranium) experience higher strains and stresses than their tall and domed counterparts (i.e. oreinirostral cranium) (McHenry et al., 2006; Rayfield and Milner, 2008; Rayfield et al., 2007). With respect to squamates, FEA performed on *Iguana* (Simões et al., 2016), *Sphenodon* (Curtis et al., 2011a), *Gekko* (Cost et al., 2020) and *Uromastyx* (Moazen et al., 2009a) predicted low stress/strain in the snout. In these taxa and *S. merianae*, the shorter, broader and somewhat domed snout probably maximise the second moment of area and thus better resist bending (Ghavami, 2015) than the long, narrow, flatter and fenestrated snout of *V. niloticus*. This might be reflected by the more dominant tensile strain in the maxilla of *V. niloticus*. However, the elongated snout of *V. niloticus* and other varanids (McCurry et al., 2015) probably increases the rotational velocity at the tip of the jaw for capturing elusive prey (Herrel et al., 2007; Metzger, 2002; Metzger and Herrel, 2005; Stayton, 2005). In addition, the lighter cranium and taller postorbital region in *V. niloticus* may maximise the rotational velocity of the head generated by the cervical muscles as varanids, with their highly specialised lingual apparatus, appear to rely more on inertial feeding than does *S. merianae* (Elias et al., 2000; Montuelle et al., 2009).

The two species studied here also differ markedly in their degree of sexual dimorphism and mating behaviours. Whereas varanids show little sexual dimorphism and males engage in ritualised, wrestling-like, combat (Khan et al., 2018; Murphy and Mitchell, 1974; Tsellarius and Tsellarius, 1997), *S. merianae* shows strong sexual dimorphism in head form and muscle size (Fabre et al., 2014a; Naretto et al., 2014), and males engage in combat involving biting. Moreover, in male *S. merianae*, bite force scales disproportionately with head width compared with that of females and is associated with more aggressive behaviours in males (Herrel et al., 2009), which, as in other dimorphic lizard species (Lailvaux and Irschick, 2007; Lappin and Husak, 2005; Lappin et al., 2006), might favour success in male combat, and resource and mate defence (Naretto et al., 2014). Accidents or antagonistic interspecific and intraspecific interactions can be associated with relatively higher bone strain and injury (Cooper and Vitt, 1987; Jurmain, 1997), which impose a greater demand for bone resistance. Strain magnitude has an important impact on the mass and distribution of bone, and variation in bone form reflects the loading regime experienced during a range of habitual and infrequent behaviours and events (Ehrlich and Lanyon, 2002; Frost, 2003; Gröning et al., 2013b; Meakin et al., 2014). Although we used a female *S. merianae* specimen, intraspecific differences were far less pronounced than those between *S. merianae* and *V. niloticus*. Therefore, it is also possible that the agonistic behaviours between males may impose a greater demand on certain regions of the cranium in *S. merianae*, resulting in lower bone strain magnitudes in the frontal and the snout, and larger gradients across the cranium compared with those in *V. niloticus* during feeding.

Our biomechanical simulations provide a mechanistic interpretation of the pattern of co-variation between bite force, cranial shape and muscle cross-sectional area observed between males and females in *S. merianae* (Fabre et al., 2014a,b). Regions of the cranium, such as the postorbital portion of the cranium and the palate, whose shape strongly co-varies with bite force and muscle cross-sectional area, are predicted to experience greater bone strain in our FEA. By contrast, cranial regions that showed little shape variation, such as the nasals, are those that experienced low bone strain. The differences in the shape of certain cranial regions between males and females in *S. merianae* might therefore represent a response to the loading regime of the whole skull, similar to the intraspecific variation shown within marsupial species due to masticatory loading (Weisbecker et al., 2019).

### The biomechanics of the frame-like cranium of lepidosaurs

We assessed the accuracy of our finite element models by comparing bone strain magnitudes calculated in a FEA series (Table 4) at individual strain gauge sites with *in vivo* bone strain measured in other squamates (Ross et al., 2018). Note that the values obtained from these additional analyses (Table 4) are not directly comparable with the strain magnitudes for the entire bones (Fig. 2B; Table S4). Strain calculated in our FEA falls within the range of values measured experimentally. Mean principal strain values measured in *Anolis equestris*, *Gekko gecko*, *Iguana iguana*, *Uromastyx geyri* and *S. merianae* ranged from 102 to 1004 με (ε_1_) and −147 to −1195 με (ε_3_) (Porro et al., 2013; Ross et al., 2018). Bone strain magnitudes calculated at strain gauge sites for *S. merianae* were underestimated when compared with published strain gauge records (Table 4) made on the same individual (with the exception of the tensile strain in the frontal), but fell within the range of those obtained on two other specimens (Ross et al., 2018). This discrepancy might be due to the fact that our simulations did not capture the whole range of loads that the cranium experiences during biting (e.g. tearing forces caused by the pull back of neck muscles, side to side shaking of relatively large prey), and/or that the restraint of the animal during transducer biting might have caused higher bone strains. With respect to *V. niloticus*, tensile strain (ε_1_) magnitudes in the frontal bone calculated in our model (Table 4) are within the range of magnitudes (100–600 με) collected by Smith and Hylander (1985) in *V. exanthematicus* during feeding. For both species, most strain magnitudes obtained from the entire bones are greater than those from strain gauge sites when similar loading cases are compared (Table S5). Together with the good match between the MDA results and experimental data, these comparisons suggest that reasonable biological interpretations can be drawn from the present biomechanical models. A validation study is currently being undertaken using unpublished *in vivo* data to further assess the accuracy of our models and determine the key parameters that affect their output.

**Table 4.**
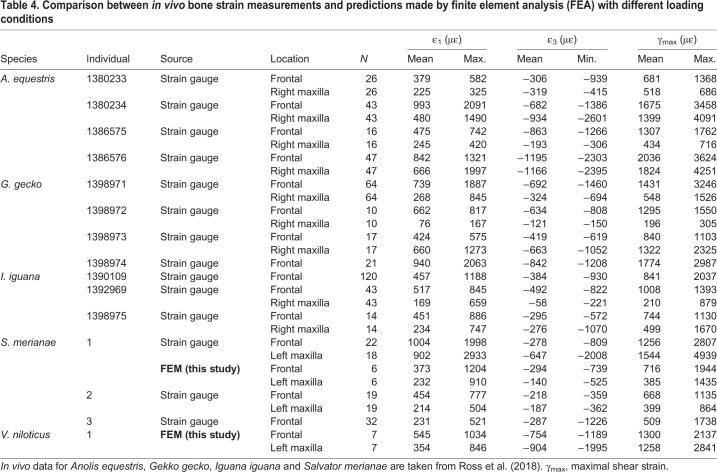
**Comparison between *in vivo* bone strain measurements and predictions made by finite element analysis (FEA) with different loading conditions**

Whether a complete postorbital bar was included or not, we observed similarities in the overall pattern and magnitude of bone strain between *S. merianae*, *V. niloticus* and other lizards. Our results thus support the hypothesis that bone strain magnitude and distribution do not radically differ between lizard species with and without a complete postorbital bar. Bone strain is not homogeneous across the cranium, and the highest strain magnitudes are located in the circumorbital and postorbital regions, and the palate (Figs 2 and 3). The pterygoid is more highly strained than any other bone, probably because it serves as an attachment area for the m. pterygoideus – the largest jaw-closing muscle. Interestingly, these regions also show high disparity and rate of evolution in lizards (Watanabe et al., 2019). Hence, biomechanical demands appear to be reflected in the variation in the form of the cranial regions at both interspecific and intraspecific levels. The parietal experiences higher strain magnitudes than the maxilla (Fig. 2) and displays large areas of peak strain whose distribution varies with the location of the bite point along the tooth row (Fig. 3). Thus, the parietal does not simply serve as a muscle attachment area to withstand muscle loads but also resists the loads transferred from the bite point to the back of the cranium. Areas of peak strain on the parietal are reduced when sutures are fused (Jones et al., 2017; Fig. S1), which further underlines the role of the parietal in resisting biting loads and the importance of sutures for load transfer across the cranium (Curtis et al., 2013; Moazen et al., 2009a). However, we do not know the relative contribution of each suture in this phenomenon and whether the fronto-parietal suture plays as prominent a role in *S. merianae* and *V. niloticus* as it does in *Uromastyx hardwickii* (Moazen et al., 2009a).

Consistent with *in vivo* strain gauge measurements, we found higher strain magnitudes in the parietal than in the maxilla when the entire bones were sampled (Fig. 2). However, the similar or higher strain magnitudes in the entire parietal compared with the entire frontal contrast with strain gauge measurements, which consistently found the opposite pattern in *Iguana*, *A. equestris* and *G. gecko* (Ross et al., 2018). This discrepancy between experimental and finite element results might be because the regions on the parietal that experience the highest strain magnitudes are covered by muscles and thus cannot be sampled with *in vivo* strain gauges (Figs 2 and 3). Although the good match between our simulations and experimental data (Tables 2 and 4) gives us confidence in our models, the effect of modelling approximations and potential artefacts still cannot be ruled out; our ongoing validation study will thus be important to clarify this point. The higher strain magnitudes in the frontals of *V. niloticus* are caused by the interfrontal suture, and it is important to note that the two species showed a consistent strain pattern across the cranial bones when this suture was fused (Fig. 2). Finally, despite marked differences in the cranial shape of the two species, the individual bones sampled across the cranium showed similar deformation regimes. Beyond species-specific differences, these results therefore suggest that lizard species with different cranial shapes may share a common deformation regime, something also seen among anthropoid primates (Ross et al., 2011).

In *S. merianae*, the postorbital bar is among the regions of the cranium that experience the highest strain magnitudes. We found that the postorbital bar was predominantly under compressive strain, whereas strain gauge measurements suggest that tension is the dominant loading regime in the postorbital bar of *U. hardwickii* (Porro et al., 2014). This difference might be because the jugal serves as an attachment area for the external bundle of the m. pterygoideus in this species. It is also worth noting that, in our models of *S. merianae*, peak tensile strain was located on the lateral side of the postorbital bar, whereas larger, peak compressive strain was on the medial side (Fig. 3). Therefore, the different deformation regimes of the postorbital bar between *S. merianae* and *U. hardwickii* could also be explained by the placement of the strain gauges on the lateral side rather than the medial of the jugal (Porro et al., 2014). Unfortunately, FEA results reported for *U. hardwickii* cannot provide clear answers to this question (Moazen et al., 2009a). Strain magnitude in the postorbital bar of *S. merianae* increased during posterior biting compared with anterior unilateral biting, as in *U. hardwickii* (Moazen et al., 2008; Porro et al., 2014), and its removal increased strain magnitude in the bones of the cranial roof and palate. When present in lizards, a complete postorbital bar therefore appears to be important for maintaining the structural integrity of the cranium by reducing its bending (during anterior biting) and twisting (during unilateral biting), and by providing an anchoring strut for the muscle attachment areas in the postorbital region.

The variation in the cranial struts of the frame-like skull of lepidosaurs was hypothesised to be tightly linked to the evolution of bite performance and feeding function (Rieppel and Gronowski, 1981). Geckos, which have lost the postorbital and supratemporal bars, have a relatively lower adductor muscle mass and bite force, and a lighter cranium compared with other squamates (Herrel et al., 2007). Although based on a limited sample, we did not find significant differences in bite force between specimens of *S. merianae* and *V. niloticus*. The loading regimes (Table 2) of the crania of the two specimens used for modelling were also similar, and *V. niloticus* skull geometry was slightly more efficient at transmitting muscle force to the bite point. Yet, the postorbital bar, digitally added in *V. niloticus*, was less efficient in decreasing peak bone strain than in *S. merianae*. Thus, neither biting performance nor the ability of the cranium to withstand high feeding loads appears to be impaired by the reduction of the postorbital bar in varanids. This suggests that morphological and/or behavioural changes during varanid evolution might compensate for the absence of a postorbital bar or reduce the importance of a previous role. We think it unlikely that the postorbital ligament alone can fulfil the role of the postorbital bar as it appears to play a minor role in strain absorption. However, our finite element model may also not represent the soft tissue anatomy adequately enough to fully exclude this possibility. In *V. niloticus*, the postorbital ligament is the thickened free anterodorsal margin of a sheet of temporal fascia that stretches across the temporal region (upper and lower fenestrae) enclosing the supratemporal and postorbital bars and attaching to the rictal fold ventrally and the quadrate posteriorly. The tensioning of muscle fascia by muscle bulging was shown to decrease peak bone strain in macaques and *Sphenodon* (Curtis et al., 2011a,b). A similar effect might occur in *V. niloticus* but including the entire temporal fascia would have greatly increased the complexity of the finite element model and analyses.

From an evolutionary perspective, the drivers of the reduction of the postorbital bar in varanids remain unclear. It is possible that the reduction of the bar was originally associated with mesokinesis that has been secondarily lost in large varanids or that the reduction of the bar is a by-product of accelerated growth of the postorbitofrontal during development (Werneburg et al., 2015). The acquisition of an active foraging life-style (McBrayer, 2004) and an inertial feeding mode (Herrel et al., 2000), which entails important accelerations of the head, have been suggested to be linked to the lengthening and lightening of crania in varanids. In this regard, the frontals of *V. niloticus* notably appear to be better optimised for maximum strength with minimal material during biting than in *S. merianae* (Figs 1 and 2). However, the available data appear to contradict this hypothesis as varanids were not found to have a lower skull to body mass ratio compared with other lepidosaurs (Metzger, 2002). Male–male interactions and mating behaviour might represent another important potential driver for cranial evolution in varanids and other lepidosaurs, but the influence of these factors on cranial mechanics has never been directly assessed.

### Cranial bone strain in lepidosaurs and other amniotes

Put in a broader context, our results bring additional insights into the factors underlying the evolution of the cranial design in amniotes. During maximal biting, the maximal shear strain magnitude recorded at the working-side postorbital bar of *S. merianae* was at least 2.5 times higher than values reported for *Eulemur*, *Otolemur*, *Aotus* and *Macaca* (Nakashige et al., 2011; Ross and Metzger, 2004; Ross et al., 2011). In *Macaca*, the postorbital bar and septum appear to have little role in the structural resistance of the cranium to biting loads (Nakashige et al., 2011; Ross et al., 2011) and might instead serve for oculomotor stability (Cartmill, 1980; Heesy et al., 2007; Nakashige et al., 2011; Ravosa et al., 2000; Ross and Hylander, 1996), whereas preliminary finite element results suggest that digitally removing the postorbital bar does impact cranial bone strain in *Eulemur* (Strait et al., 2014). Bones forming the circumorbital region in *S. merianae* and *V. niloticus* also experienced higher peak strain magnitude than in the homologous region in mammals (Bright, 2012; Ross and Metzger, 2004; Ross et al., 2011). These differences might reflect the greater biomechanical role of the circumorbital region during biting in lizards compared with mammals.

Strain distribution across the cranial bones of *S. merianae* and *V. niloticus* is more homogeneous that in mammals. Consistent with previous observations made on lizards, the calvarial bones (parietal and frontal) of *S. merianae* and *V. niloticus* have higher strain magnitudes than those recorded in mammals. The parietal is more loaded than the maxilla in lizards, whereas it experiences lower strain than the facial bones in mammals (Behrents et al., 1978; Bright, 2012; Cox et al., 2012; Herring and Teng, 2000; Ross and Metzger, 2004; Thomason et al., 2001). Previous studies (Cox et al., 2012) and our preliminary results also suggest lower strain gradients and magnitudes in the palate of rodents and rabbits compared with values in the two lizards studied here. However, a more detailed comparison with finite element results obtained on mammals is difficult as previous analyses did not necessarily incorporate the same level of details or employ the same metrics. The combination of anatomical, developmental and biomechanical data in an explicit phylogenetic framework will be essential to better understand the determinants of the variation in the skull form during tetrapod evolution.

### Conclusions

We used *in vivo* bite force measurements, high-speed X-ray videoradiography and computer-based biomechanical simulation techniques to investigate the cranial biomechanics of *S. merianae* and *V. niloticus*. The differences in the strain regimes of the cranial bones were not related to the presence of a complete postorbital bar, but rather to the distinct overall cranial architecture observed between these two species (tall and broad snout in *S. merianae*, long and narrow snout in *V. niloticus*). The postorbital bar is important for the structural resistance of the cranium to feeding loads in *S. merianae*, and potentially during antagonist male–male interactions, whereas the postorbital ligament probably does not have a substantial biomechanical role in *V. niloticus*. Our results suggest that the reduction of the postorbital bar in *V. niloticus* impaired neither its biting performance nor the structural resistance of the cranium to feeding loads. Beyond differences related to species-specific variation in morphology, the two species share a similar strain and deformation regime of the cranium during biting. Strain magnitude is greater in the postorbital region (specifically in the parietal and pterygoid) and the circumorbital region, which appears to be important for resisting feeding loads. This suggests that common mechanical behaviour might underlie the frame-like cranium of lizards.

## References

[JEB234831C1] Ahrens, J., Geveci, B. and Law, C. (2005). ParaView: an end-user tool for large-data visualization. In *Visualization Handbook* (ed. C. R. Johnson), pp. 717-731. Burlington, USA: Butterworth-Heinemann.

[JEB234831C2] Behrents, R. G., Carlson, D. S. and Abdelnour, T. (1978). *In vivo* analysis of bone strain about the sagittal suture in *Macaca mulatta* during masticatory movements. *J. Dent. Res.* 57, 904-908. 10.1177/00220345780570091401 102671

[JEB234831C3] Bright, J. A. (2012). The importance of craniofacial sutures in biomechanical finite element models of the domestic pig. *PLoS ONE* 7, e31769. 10.1371/journal.pone.0031769 22363727 PMC3283651

[JEB234831C4] Burbrink, F. T., Grazziotin, F. G., Pyron, R. A., Cundall, D., Donnellan, S., Irish, F., Keogh, J. S., Kraus, F., Murphy, R. W., Noonan, B. et al. (2020). Interrogating genomic-scale data for Squamata (lizards, snakes, and amphisbaenians) shows no support for key traditional morphological relationships. *Syst. Biol.* 69, 502-520. 10.1093/sysbio/syz062 31550008

[JEB234831C5] Cartmill, M. (1980). Morphology, function, and evolution of the anthropoid postorbital septum. In *Evolutionary Biology of the New World Monkeys and Continental Drift* (ed. R. L. Ciochon and A. B. Chiarelli), pp. 243-274. Boston, USA: Springer US.

[JEB234831C6] Colli, G. R., Péres, A. K. and da Cunha, H. J. (1998). A new species of *Tupinambis* (Squamata: Teiidae) from central Brazil, with an analysis of morphological and genetic variation in the genus. *Herpetologica* 54, 477-492.

[JEB234831C7] Cooper, W. E. Jr. and Vitt, L. J. (1987). Deferred agonistic behavior in a long-lived scincid lizard *Eumeces laticeps*. *Oecologia* 72, 321-326. 10.1007/BF00377558 28311124

[JEB234831C8] Cost, I. N., Middleton, K. M., Sellers, K. C., Echols, M. S., Witmer, L. M., Davis, J. L. and Holliday, C. M. (2020). Palatal biomechanics and its significance for cranial kinesis in *Tyrannosaurus rex*. *Anat. Rec.* 303, 999-1017. 10.1002/ar.24219 31260190

[JEB234831C9] Cox, P. G., Rayfield, E. J., Fagan, M. J., Herrel, A., Pataky, T. C. and Jeffery, N. (2012). Functional evolution of the feeding system in rodents. *PLoS ONE* 7, e36299. 10.1371/journal.pone.0036299 22558427 PMC3338682

[JEB234831C10] Curtis, N., Jones, M. E. H., Evans, S. E., Shi, J., O'Higgins, P. and Fagan, M. J. (2010). Predicting muscle activation patterns from motion and anatomy: modelling the skull of Sphenodon (Diapsida: Rhynchocephalia). *J. R. Soc. Interface* 7, 153-160. 10.1098/rsif.2009.0139 19474084 PMC2839385

[JEB234831C11] Curtis, N., Jones, M. E. H., Shi, J., O'Higgins, P., Evans, S. E. and Fagan, M. J. (2011a). Functional relationship between skull form and feeding mechanics in Sphenodon, and implications for Diapsid skull development. *PLoS ONE* 6, e29804. 10.1371/journal.pone.0029804 22216358 PMC3247290

[JEB234831C12] Curtis, N., Witzel, U., Fitton, L., O'Higgins, P. and Fagan, M. (2011b). The mechanical significance of the temporal fasciae in *Macaca fascicularis*: an investigation using finite element analysis. *Anat. Rec.* 294, 1178-1190. 10.1002/ar.21415 21618443

[JEB234831C13] Curtis, N., Jones, M. E. H., Evans, S. E., O'Higgins, P. and Fagan, M. J. (2013). Cranial sutures work collectively to distribute strain throughout the reptile skull. *J. R. Soc. Interface* 10, 20130442. 10.1098/rsif.2013.0442 23804444 PMC3730698

[JEB234831C14] Daudin, F. M. (1802). *Histoire Générale et Particulière des Reptiles*. Paris, France: Imprimerie Dufart.

[JEB234831C15] Dowell, S. A., Portik, D. M., de Buffrénil, V., Ineich, I., Greenbaum, E., Kolokotronis, S.-O. and Hekkala, E. R. (2016). Molecular data from contemporary and historical collections reveal a complex story of cryptic diversification in the *Varanus* (*Polydaedalus*) *niloticus* species group. *Mol. Phylogenet. Evol.* 94, 591-604. 10.1016/j.ympev.2015.10.004 26475616

[JEB234831C16] Ehrlich, P. J. and Lanyon, L. E. (2002). Mechanical strain and bone cell function: a review. *Osteoporos. Int.* 13, 688-700. 10.1007/s001980200095 12195532

[JEB234831C17] Elias, J. A., McBrayer, L. D. and Reilly, S. M. (2000). Prey transport kinematics in *Tupinambis teguixin* and *Varanus exanthematicus*: conservation of feeding behavior in ‘chemosensory-tongued’ lizards. *J. Exp. Biol.* 203, 791-801. 10648221 10.1242/jeb.203.4.791

[JEB234831C18] Evans, S. E. (2008). The skull of lizards and tuatara. In *Biology of the Reptilia, The Skull of Lepidosauria* (ed. C. Gans A. S. Gaunt and K. Adler), pp. 1-347. Ithaca, USA: Society for the Study of Amphibians and Reptiles.

[JEB234831C19] Fabre, A.-C., Andrade, D. V., Huyghe, K., Cornette, R. and Herrel, A. (2014a). Interrelationships between bones, muscles, and performance: biting in the lizard *Tupinambis merianae*. *Evol. Biol.* 41, 518-527. 10.1007/s11692-014-9286-3

[JEB234831C20] Fabre, A.-C., Cornette, R., Huyghe, K., Andrade, D. V. and Herrel, A. (2014b). Linear versus geometric morphometric approaches for the analysis of head shape dimorphism in lizards. *J. Morphol.* 275, 1016-1026. 10.1002/jmor.20278 24740578

[JEB234831C21] Fenech, C. M. and Keaveny, T. M. (1999). A cellular solid criterion for predicting the axial-shear failure properties of bovine trabecular bone. *J. Biomech. Eng.* 121, 414-422. 10.1115/1.2798339 10464696

[JEB234831C22] Frazzetta, T. H. (1962). A functional consideration of cranial kinesis in lizards. *J. Morphol.* 111, 287-319. 10.1002/jmor.1051110306 13959380

[JEB234831C23] Frost, H. M. (2003). Bone's mechanostat: a 2003 update. *Anat. Rec.* 275A, 1081-1101. 10.1002/ar.a.10119 14613308

[JEB234831C24] Gans, C. (1982). Fiber architecture and muscle function. *Exerc. Sport Sci. Rev.* 10, 160-207. 10.1249/00003677-198201000-00006 6749514

[JEB234831C25] Ghavami, P. (2015). *Mechanics of Materials*. Cham, Switzerland: Springer International Publishing.

[JEB234831C26] Gröning, F., Jones, M. E. H., Curtis, N., Herrel, A., O'Higgins, P., Evans, S. E. and Fagan, M. J. (2013a). The importance of accurate muscle modelling for biomechanical analyses: a case study with a lizard skull. *J. R. Soc. Interface* 10, 20130216. 10.1098/rsif.2013.0216 23614944 PMC3673157

[JEB234831C27] Gröning, F., Fagan, M. and O'Higgins, P. (2013b). Comparing the distribution of strains with the distribution of bone tissue in a human mandible: a finite element study. *Anat. Rec.* 296, 9-18. 10.1002/ar.22597 22976999

[JEB234831C28] Heesy, C. P., Ross, C. F. and Demes, B. (2007). Oculomotor stability and the functions of the postorbital bar and septum. In *Primate Origins: Adaptations and Evolution* (ed. M. J. Ravosa and M. Dagosto), pp. 257-283. Boston, USA: Springer US.

[JEB234831C29] Herrel, A., De Vree, F., Delheusy, V. and Gans, C. (1999a). Cranial kinesis in gekkonid lizards. *J. Exp. Biol.* 202, 3687-3698. 10574746 10.1242/jeb.202.24.3687

[JEB234831C30] Herrel, A., Spithoven, L., Van Damme, R. and De Vree, F. (1999b). Sexual dimorphism of head size in *Gallotia galloti*: testing the niche divergence hypothesis by functional analyses. *Funct. Ecol.* 13, 289-297. 10.1046/j.1365-2435.1999.00305.x

[JEB234831C31] Herrel, A., Aerts, P. and De Vree, F. (2000). Cranial kinesis in geckoes: functional implications. *J. Exp. Biol.* 203, 1415-1423. 10751157 10.1242/jeb.203.9.1415

[JEB234831C32] Herrel, A., Schaerlaeken, V., Meyers, J. J., Metzger, K. A. and Ross, C. F. (2007). The evolution of cranial design and performance in squamates: consequences of skull-bone reduction on feeding behavior. *Integr. Comp. Biol.* 47, 107-117. 10.1093/icb/icm014 21672824

[JEB234831C33] Herrel, A., Andrade, D. V., de Carvalho, J. E., Brito, A., Abe, A. and Navas, C. (2009). Aggressive behavior and performance in the tegu lizard *Tupinambis merianae*. *Physiol. Biochem. Zool.* 82, 680-685. 10.1086/605935 19758090

[JEB234831C34] Herring, S. W. and Teng, S. (2000). Strain in the braincase and its sutures during function. *Am. J. Phys. Anthropol.* 112, 575-593. 10.1002/1096-8644(200008)112:4<575::AID-AJPA10>3.0.CO;2-0 10918130 PMC2813197

[JEB234831C35] Hylander, W. L. and Johnson, K. R. (1997). *In vivo* bone strain patterns in the zygomatic arch of macaques and the significance of these patterns for functional interpretations of craniofacial form. *Am. J. Phys. Anthropol.* 102, 203-232. 10.1002/(SICI)1096-8644(199702)102:2<203::AID-AJPA5>3.0.CO;2-Z 9066901

[JEB234831C36] Hylander, W. L., Picq, P. G. and Johnson, K. R. (1991). Function of the supraorbital region of primates. *Arch. Oral Biol.* 36, 273-281. 10.1016/0003-9969(91)90097-E 2064549

[JEB234831C37] Jones, M. E. H., Curtis, N., Fagan, M. J., O'Higgins, P. and Evans, S. E. (2011). Hard tissue anatomy of the cranial joints in *Sphenodon* (Rhynchocephalia): sutures, kinesis, and skull mechanics. *Palaeontol. Electron.* 14, 17A.

[JEB234831C38] Jones, M. E. H., Gröning, F., Dutel, H., Sharp, A., Fagan, M. J. and Evans, S. E. (2017). The biomechanical role of the chondrocranium and sutures in a lizard cranium. *J. R. Soc. Interface* 14, 20170637. 10.1098/rsif.2017.0637 29263126 PMC5746569

[JEB234831C39] Jurmain, R. (1997). Skeletal evidence of trauma in African apes, with special reference to the gombe chimpanzees. *Primates* 38, 1-14. 10.1007/BF02385918

[JEB234831C40] Khan, A. R., Alam, S. M. I., Islam, J., Hasan, S., Salahuddin Mia, R., Mia, J., Khandakar, N. and Parves, N. (2018). An observational note on mating behavior and male-male combat of the Bengal monitor *Varanus bengalensis* (Daudin, 1802) in the National Botanical Garden, Dhaka. *Biawak* 12, 100-103.

[JEB234831C41] Lailvaux, S. P. and Irschick, D. J. (2007). The evolution of performance-based male fighting ability in Caribbean *Anolis* lizards. *Am. Nat.* 170, 573-586. 10.1086/521234 17891736

[JEB234831C42] Lappin, A. K. and Husak, J. F. (2005). Weapon performance, not size, determines mating success and potential reproductive output in the collared lizard (*Crotaphytus collaris*). *Am. Nat.* 166, 426-436. 10.1086/432564 16224696

[JEB234831C43] Lappin, A. K. and Jones, M. E. H. (2014). Reliable quantification of bite-force performance requires use of appropriate biting substrate and standardization of bite out-lever. *J. Exp. Biol.* 217, 4303-4312. 10.1242/jeb.106385 25359934

[JEB234831C44] Lappin, A. K., Hamilton, P. S. and Sullivan, B. K. (2006). Bite-force performance and head shape in a sexually dimorphic crevice-dwelling lizard, the common chuckwalla [*Sauromalus ater* (= *obesus*)]. *Biol. J. Linn. Soc.* 88, 215-222. 10.1111/j.1095-8312.2006.00615.x

[JEB234831C45] Lautenschlager, S., Witmer, L. M., Altangerel, P. and Rayfield, E. J. (2013). Edentulism, beaks, and biomechanical innovations in the evolution of theropod dinosaurs. *Proc. Natl. Acad. Sci. USA* 110, 20657-20662. 10.1073/pnas.1310711110 24297877 PMC3870693

[JEB234831C46] Luiselli, L., Akani, G. C. and Capizzi, D. (1999). Is there any interspecific competition between dwarf crocodiles (*Osteolaemus tetraspis*) and Nile monitors (*Varanus niloticus ornatus*) in the swamps of central Africa? A study from south-eastern Nigeria. *J. Zool.* 247, 127-131. 10.1111/j.1469-7998.1999.tb00200.x

[JEB234831C47] McBrayer, L. D. (2004). The relationship between skull morphology, biting performance and foraging mode in Kalahari lacertid lizards. *Zool. J. Linn. Soc.* 140, 403-416. 10.1111/j.1096-3642.2003.00106.x

[JEB234831C48] McCurry, M. R., Mahony, M., Clausen, P. D., Quayle, M. R., Walmsley, C. W., Jessop, T. S., Wroe, S., Richards, H. and McHenry, C. R. (2015). The relationship between cranial structure, biomechanical performance and ecological diversity in varanoid lizards. *PLoS ONE* 10, e0130625. 10.1371/journal.pone.0130625 26106889 PMC4479569

[JEB234831C49] McHenry, C. R., Clausen, P. D., Daniel, W. J. T., Meers, M. B. and Pendharkar, A. (2006). Biomechanics of the rostrum in crocodilians: a comparative analysis using finite-element modeling. *Anat. Rec.* 288A, 827-849. 10.1002/ar.a.20360 16835925

[JEB234831C50] Meakin, L. B., Price, J. S. and Lanyon, L. E. (2014). The contribution of experimental *in vivo* models to understanding the mechanisms of adaptation to mechanical loading in bone. *Front. Endocrinol.* 5, 154. 10.3389/fendo.2014.00154 PMC4181237 25324829

[JEB234831C51] Mendez, J. and Keys, A. (1960). Density and composition of mammalian muscle. *Metabolism* 9, 184-188.

[JEB234831C52] Metzger, K. A. (2002). Cranial kinesis in lepidosaurs: skulls in motion. In *Topics in Functional and Ecological Vertebrate Morphology* (ed. P. Aerts, K. D'Aout, A. Herrel and R. Van Damme), pp. 205-236. Maastricht, Netherlands: Shaker Publishing.

[JEB234831C53] Metzger, K. A. and Herrel, A. (2005). Correlations between lizard cranial shape and diet: a quantitative, phylogenetically informed analysis. *Biol. J. Linn. Soc.* 86, 433-466. 10.1111/j.1095-8312.2005.00546.x

[JEB234831C54] Moazen, M., Curtis, N., Evans, S. E., O'Higgins, P. and Fagan, M. J. (2008). Combined finite element and multibody dynamics analysis of biting in a *Uromastyx hardwickii* lizard skull. *J. Anat.* 213, 499-508. 10.1111/j.1469-7580.2008.00980.x 19014357 PMC2667544

[JEB234831C55] Moazen, M., Curtis, N., O'Higgins, P., Jones, M. E. H., Evans, S. E. and Fagan, M. J. (2009a). Assessment of the role of sutures in a lizard skull: a computer modelling study. *Proc. R. Soc. B Biol. Sci.* 276, 39-46. 10.1098/rspb.2008.0863 PMC2614251 18765341

[JEB234831C56] Moazen, M., Curtis, N., O'Higgins, P., Evans, S. E. and Fagan, M. J. (2009b). Biomechanical assessment of evolutionary changes in the lepidosaurian skull. *Proc. Natl. Acad. Sci.* 106, 8273-8277. 10.1073/pnas.0813156106 19416822 PMC2688846

[JEB234831C57] Montuelle, S. J. and Williams, S. H. (2015). *In vivo* measurement of mesokinesis in *Gekko gecko*: the role of cranial kinesis during gape display, feeding and biting. *PLoS ONE* 10, e0134710. 10.1371/journal.pone.0134710 26230087 PMC4521707

[JEB234831C58] Montuelle, S. J., Herrel, A., Schaerlaeken, V., Metzger, K. A., Mutuyeyezu, A. and Bels, V. L. (2009). Inertial feeding in the teiid lizard *Tupinambis merianae*: the effect of prey size on the movements of hyolingual apparatus and the cranio-cervical system. *J. Exp. Biol.* 212, 2501-2510. 10.1242/jeb.026336 19648393

[JEB234831C59] Munns, S. W., Jayaraman, G. and Luallin, S. R. (1994). Effects of pretwist on biomechanical properties of canine patellar tendon. *Arthrosc. J. Arthrosc. Relat. Surg.* 10, 404-411. 10.1016/S0749-8063(05)80191-4 7945636

[JEB234831C60] Murphy, J. B. and Mitchell, L. A. (1974). Ritualized combat behavior of the pygmy mulga monitor lizard, *Varanus gilleni* (Sauria: Varanidae). *Herpetologica* 30, 90-97.

[JEB234831C61] Nakagawa, Y., Hayashi, K., Yamamoto, N. and Nagashima, K. (1996). Age-related changes in biomechanical properties of the Achilles tendon in rabbits. *Eur. J. Appl. Physiol. Occup. Physiol.* 73, 7-10. 10.1007/BF00262803 8861663

[JEB234831C62] Nakashige, M., Smith, A. L. and Strait, D. S. (2011). Biomechanics of the macaque postorbital septum investigated using finite element analysis: implications for anthropoid evolution. *J. Anat.* 218, 142-150. 10.1111/j.1469-7580.2010.01316.x 21070237 PMC3039786

[JEB234831C63] Nalla, R. K., Kinney, J. H. and Ritchie, R. O. (2003). Mechanistic fracture criteria for the failure of human cortical bone. *Nat. Mater.* 2, 164-168. 10.1038/nmat832 12612673

[JEB234831C64] Naretto, S., Cardozo, G., Blengini, C. S. and Chiaraviglio, M. (2014). Sexual selection and dynamics of jaw muscle in *Tupinambis* lizards. *Evol. Biol.* 41, 192-200. 10.1007/s11692-013-9257-0

[JEB234831C65] Patchell, F. C. and Shine, R. (1986). Feeding mechanisms in pygopodid lizards: how can Lialis swallow such large prey? *J. Herpetol.* 20, 59-64. 10.2307/1564125

[JEB234831C66] Porro, L. B., Metzger, K. A., Iriarte-Diaz, J. and Ross, C. F. (2013). *In vivo* bone strain and finite element modeling of the mandible of *Alligator mississippiensis*. *J. Anat.* 223, 195-227. 10.1111/joa.12080 23855772 PMC3972043

[JEB234831C67] Porro, L. B., Ross, C. F., Iriarte-Diaz, J., O'Reilly, J. C., Evans, S. E. and Fagan, M. J. (2014). *In vivo cranial* bone strain and bite force in the agamid lizard *Uromastyx geyri*. *J. Exp. Biol.* 217, 1983-1992. 10.1242/jeb.096362 24577443 PMC4059540

[JEB234831C68] Preuschoft, H. and Witzel, U. (2002). Biomechanical investigations on the skulls of reptiles and mammals. *Senckenb. Lethaea* 82, 207. 10.1007/BF03043785

[JEB234831C69] Pyron, R. A. (2017). Novel approaches for phylogenetic inference from morphological data and total-evidence dating in squamate reptiles (lizards, snakes, and amphisbaenians). *Syst. Biol.* 66, 38-56. 10.1093/sysbio/syw068 28173602

[JEB234831C70] Ravosa, M. J., Noble, V. E., Hylander, W. L., Johnson, K. R. and Kowalski, E. M. (2000). Masticatory stress, orbital orientation and the evolution of the primate postorbital bar. *J. Hum. Evol.* 38, 667-693. 10.1006/jhev.1999.0380 10799259

[JEB234831C71] Rayfield, E. J. and Milner, A. C. (2008). Establishing a framework for archosaur cranial mechanics. *Paleobiology* 34, 494-515. 10.1666/07006.1

[JEB234831C72] Rayfield, E. J., Milner, A. C., Xuan, V. B. and Young, P. G. (2007). Functional morphology of spinosaur ‘crocodile-mimic’ dinosaurs. *J. Vertebr. Paleontol.* 27, 892-901. 10.1671/0272-4634(2007)27[892:FMOSCD]2.0.CO;2

[JEB234831C73] Reed, D. A., Porro, L. B., Iriarte-Diaz, J., Lemberg, J. B., Holliday, C. M., Anapol, F. and Ross, C. F. (2011). The impact of bone and suture material properties on mandibular function in *Alligator mississippiensis*: testing theoretical phenotypes with finite element analysis. *J. Anat.* 218, 59-74. 10.1111/j.1469-7580.2010.01319.x 21091693 PMC3039781

[JEB234831C74] Rieppel, O. (1978). Streptostyly and muscle function in lizards. *Experimentia* 34, 776-777. 10.1007/BF01947321

[JEB234831C75] Rieppel, O. and Gronowski, R. W. (1981). The loss of the lower temporal arcade in diapsid reptiles. *Zool. J. Linn. Soc.* 72, 203-217. 10.1111/j.1096-3642.1981.tb01570.x

[JEB234831C76] Ross, C. F. (2001). *In vivo* function of the craniofacial haft: the interorbital “pillar*”*. *Am. J. Phys. Anthropol.* 116, 108-139. 10.1002/ajpa.1106 11590585

[JEB234831C77] Ross, C. F. and Hylander, W. L. (1996). *In vivo* and *in vitro* bone strain in the owl monkey circumorbital region and the function of the postorbital septum. *Am. J. Phys. Anthropol.* 101, 183-215. 10.1002/(SICI)1096-8644(199610)101:2<183::AID-AJPA6>3.0.CO;2-3 8893085

[JEB234831C78] Ross, C. F. and Metzger, K. A. (2004). Bone strain gradients and optimization in vertebrate skulls. *Ann. Anat. Anat. Anzeiger* 186, 387-396. 10.1016/S0940-9602(04)80070-0 15646269

[JEB234831C79] Ross, C. F., Berthaume, M. A., Dechow, P. C., Iriarte-Diaz, J., Porro, L. B., Richmond, B. G., Spencer, M. and Strait, D. (2011). *In vivo* bone strain and finite-element modeling of the craniofacial haft in catarrhine primates. *J. Anat.* 218, 112-141. 10.1111/j.1469-7580.2010.01322.x 21105871 PMC3039785

[JEB234831C80] Ross, C. F., Porro, L. B., Herrel, A., Evans, S. E. and Fagan, M. J. (2018). Bite force and cranial bone strain in four species of lizards. *J. Exp. Biol.* 221, jeb180240. 10.1242/jeb.180240 30352826

[JEB234831C81] Sacks, R. D. and Roy, R. R. (1982). Architecture of the hind limb muscles of cats: functional significance. *J. Morphol.* 173, 185-195. 10.1002/jmor.1051730206 7120421

[JEB234831C82] Schaerlaeken, V., Montuelle, S. J., Aerts, P. and Herrel, A. (2011). Jaw and hyolingual movements during prey transport in varanid lizards: effects of prey type. *Zoology* 114, 165-170. 10.1016/j.zool.2010.11.008 21600748

[JEB234831C83] Schileo, E., Taddei, F., Cristofolini, L. and Viceconti, M. (2008). Subject-specific finite element models implementing a maximum principal strain criterion are able to estimate failure risk and fracture location on human femurs tested *in vitro*. *J. Biomech.* 41, 356-367. 10.1016/j.jbiomech.2007.09.009 18022179

[JEB234831C84] Schindelin, J., Arganda-Carreras, I., Frise, E., Kaynig, V., Longair, M., Pietzsch, T., Preibisch, S., Rueden, C., Saalfeld, S., Schmid, B. et al. (2012). Fiji: an open-source platform for biological-image analysis. *Nat. Methods* 9, 676. 10.1038/nmeth.2019 22743772 PMC3855844

[JEB234831C85] Sellers, W. I. and Crompton, R. H. (2004). Using sensitivity analysis to validate the predictions of a biomechanical model of bite forces. *Ann. Anat. - Anat. Anzeiger* 186, 89-95. 10.1016/S0940-9602(04)80132-8 14994917

[JEB234831C86] Sharp, A. C. and Rich, T. H. (2016). Cranial biomechanics, bite force and function of the endocranial sinuses in *Diprotodon optatum*, the largest known marsupial. *J. Anat.* 228, 984-995. 10.1111/joa.12456 26939052 PMC5341585

[JEB234831C87] Shetye, S. S., Malhotra, K., Ryan, S. D. and Puttlitz, C. M. (2009). Determination of mechanical properties of canine carpal ligaments. *Am. J. Vet. Res.* 70, 1026-1030. 10.2460/ajvr.70.8.1026 19645585

[JEB234831C88] Simões, T. R., Funston, G. F., Vafaeian, B., Nydam, R. L., Doschak, M. R. and Caldwell, M. W. (2016). Reacquisition of the lower temporal bar in sexually dimorphic fossil lizards provides a rare case of convergent evolution. *Sci. Rep.* 6, 24087. 10.1038/srep24087 27071447 PMC4829860

[JEB234831C90] Smith, K. K. and Hylander, W. L. (1985). Strain gauge measurement of mesokinetic movement in the lizard *Varanus exanthematicus*. *J. Exp. Biol.* 114, 53-70. 4009109 10.1242/jeb.114.1.53

[JEB234831C91] Stäubli, H. U., Schatzmann, L., Brunner, P., Rincón, L. and Nolte, L.-P. (1999). Mechanical tensile properties of the quadriceps tendon and patellar ligament in young adults. *Am. J. Sports Med.* 27, 27-34. 10.1177/03635465990270011301 9934415

[JEB234831C92] Stayton, C. T. (2005). Morphological evolution of the lizard skull: a geometric morphometrics survey. *J. Morphol.* 263, 47-59. 10.1002/jmor.10288 15536647

[JEB234831C93] Stayton, C. T. (2006). Testing hypotheses of convergence with multivariate data: morphological and functional convergence among herbivorous lizards. *Evolution* 60, 824-841. 10.1111/j.0014-3820.2006.tb01160.x 16739463

[JEB234831C94] Stayton, C. T. (2008). Is convergence surprising? An examination of the frequency of convergence in simulated datasets. *J. Theor. Biol.* 252, 1-14. 10.1016/j.jtbi.2008.01.008 18321532

[JEB234831C95] Stayton, C. T. (2011). Biomechanics on the half shell: functional performance influences patterns of morphological variation in the emydid turtle carapace. *Zoology* 114, 213-223. 10.1016/j.zool.2011.03.002 21820295

[JEB234831C96] Strait, D. S., Parisi, D., Sohnen, S., Smith, A. L., Tamvada, K. H., Ledogar, J. A., Ross, C. F. and Ryan, T. M. (2014). Biomechanics of the postorbital bar of *Eulemur fulvus* examined using finite element analysis. *Am. J. Phys. Anthropol.* 143, 247.

[JEB234831C97] Thomason, J. J., Grovum, L. E., Deswysen, A. G. and Bignell, W. W. (2001). *In vivo* surface strain and stereology of the frontal and maxillary bones of sheep: implications for the structural design of the mammalian skull. *Anat. Rec.* 264, 325-338. 10.1002/ar.10025 11745088

[JEB234831C98] Tonini, J. F. R., Beard, K. H., Ferreira, R. B., Jetz, W. and Pyron, R. A. (2016). Fully-sampled phylogenies of squamates reveal evolutionary patterns in threat status. *Biol. Conserv.* 204, 23-31. 10.1016/j.biocon.2016.03.039

[JEB234831C99] Tsellarius, A. and Tsellarius, E. Y. (1997). Behavior of *Varanus griseus* during encounters with conspecifics. *Asiat. Herpetol. Res.* 7, 108-130.

[JEB234831C100] Vafek, E. C., Plate, J. F., Friedman, E., Mannava, S., Scott, A. T. and Danelson, K. A. (2018). The effect of strain and age on the mechanical properties of rat Achilles tendons. *Muscles Ligaments Tendons J.* 7, 548-553. 10.32098/mltj.03.2017.19 29387650 PMC5774930

[JEB234831C101] Watanabe, A., Fabre, A.-C., Felice, R. N., Maisano, J. A., Müller, J., Herrel, A. and Goswami, A. (2019). Ecomorphological diversification in squamates from conserved pattern of cranial integration. *Proc. Natl. Acad. Sci. USA* 116, 14688-14697. 10.1073/pnas.1820967116 31262818 PMC6642379

[JEB234831C102] Weisbecker, V., Guillerme, T., Speck, C., Sherratt, E., Abraha, H. M., Sharp, A. C., Terhune, C. E., Collins, S., Johnston, S. and Panagiotopoulou, O. (2019). Individual variation of the masticatory system dominates 3D skull shape in the herbivory-adapted marsupial wombats. *Front. Zool.* 16, 41. 10.1186/s12983-019-0338-5 31695725 PMC6824091

[JEB234831C103] Werneburg, I., Polachowski, K. M. and Hutchinson, M. N. (2015). Bony skull development in the Argus monitor (Squamata, Varanidae, *Varanus panoptes*) with comments on developmental timing and adult anatomy. *Zoology* 118, 255-280. 10.1016/j.zool.2015.02.004 26051699

[JEB234831C104] Werner, Y. L. and Seifan, T. (2006). Eye size in geckos: asymmetry, allometry, sexual dimorphism, and behavioral correlates. *J. Morphol.* 267, 1486-1500. 10.1002/jmor.10499 17117406

[JEB234831C105] Wilken, A. T., Middleton, K. M., Sellers, K. C., Cost, I. N. and Holliday, C. M. (2019). The roles of joint tissues and jaw muscles in palatal biomechanics of the savannah monitor (*Varanus exanthematicus*) and their significance for cranial kinesis. *J. Exp. Biol.* 222, jeb201459. 10.1242/jeb.201459 31481636

[JEB234831C106] Yosibash, Z., Tal, D. and Trabelsi, N. (2010). Predicting the yield of the proximal femur using high-order finite-element analysis with inhomogeneous orthotropic material properties. *Philos. Trans. R. Soc. A Math. Phys. Eng. Sci.* 368, 2707-2723. 10.1098/rsta.2010.0074 20439270

